# Engagement With the Centers for Disease Control and Prevention Coronavirus Self-Checker and Guidance Provided to Users in the United States From March 23, 2020, to April 19, 2021: Thematic and Trend Analysis

**DOI:** 10.2196/39054

**Published:** 2023-03-10

**Authors:** Ami B Shah, Eghosa Oyegun, William Brett Hampton, Antonio Neri, Nicole Maddox, Danielle Raso, Paramjit Sandhu, Anita Patel, Lisa M Koonin, Leslie Lee, Lauren Roper, Geoffrey Whitfield, David A Siegel, Emily H Koumans

**Affiliations:** 1 Centers for Disease Control and Prevention, COVID-19 Emergency Response Atlanta, GA United States; 2 General Dynamics Information Technology Falls Church, VA United States; 3 Abt Associates Rockville, MD United States; 4 Health Preparedness Partners Atlanta, GA United States

**Keywords:** COVID-19, automated symptom checker, Self-Checker, triage, medical care, online information seeking, clinical assessment tool

## Abstract

**Background:**

In 2020, at the onset of the COVID-19 pandemic, the United States experienced surges in healthcare needs, which challenged capacity throughout the healthcare system. Stay-at-home orders in many jurisdictions, cancellation of elective procedures, and closures of outpatient medical offices disrupted patient access to care. To inform symptomatic persons about when to seek care and potentially help alleviate the burden on the healthcare system, Centers for Disease Control and Prevention (CDC) and partners developed the CDC Coronavirus Self-Checker (“Self-Checker”). This interactive tool assists individuals seeking information about COVID-19 to determine the appropriate level of care by asking demographic, clinical, and nonclinical questions during an online “conversation.”

**Objective:**

This paper describes user characteristics, trends in use, and recommendations delivered by the Self-Checker between March 23, 2020, and April 19, 2021, for pursuing appropriate levels of medical care depending on the severity of user symptoms.

**Methods:**

User characteristics and trends in completed conversations that resulted in a care message were analyzed. Care messages delivered by the Self-Checker were manually classified into three overarching conversation themes: (1) seek care immediately; (2) take no action, or stay home and self-monitor; and (3) conversation redirected. Trends in 7-day averages of conversations and COVID-19 cases were examined with development and marketing milestones that potentially impacted Self-Checker user engagement.

**Results:**

Among 16,718,667 completed conversations, the Self-Checker delivered recommendations for 69.27% (n=11,580,738) of all conversations to “take no action, or stay home and self-monitor”; 28.8% (n=4,822,138) of conversations to “seek care immediately”; and 1.89% (n=315,791) of conversations were redirected to other resources without providing any care advice. Among 6.8 million conversations initiated for self-reported sick individuals without life-threatening symptoms, 59.21% resulted in a recommendation to “take no action, or stay home and self-monitor.” Nearly all individuals (99.8%) who were not sick were also advised to “take no action, or stay home and self-monitor.”

**Conclusions:**

The majority of Self-Checker conversations resulted in advice to take no action, or stay home and self-monitor. This guidance may have reduced patient volume on the medical system; however, future studies evaluating patients’ satisfaction, intention to follow the care advice received, course of action, and care modality pursued could clarify the impact of the Self-Checker and similar tools during future public health emergencies.

## Introduction

In 2020, at the onset of the COVID-19 pandemic, the United States experienced surges in healthcare needs that challenged patient care capacity across all levels of the healthcare system. Stay-at-home orders in many jurisdictions, cancellations of elective procedures, and closures of outpatient medical offices disrupted patient access to care. To reduce the burden on healthcare providers, frontline organizations worked to address the patient surge by using web- and app-based approaches such as symptom-checking tools that use artificial intelligence to guide patients to appropriate levels of care [1]. One such tool is the Coronavirus Self-Checker (hereafter “Self-Checker”), which was developed and launched in spring 2020 by Centers for Disease Control and Prevention (CDC) and external partners [2]. The Self-Checker’s content and triage decision tree were adapted from protocols originally developed by CDC in 2013 for pandemic influenza planning efforts [3].

The Self-Checker is an interactive assessment tool that assists individuals aged 13 years and older, and parents and caregivers of children aged 2 to 12 years on deciding when to seek testing or medical care if they suspect that they or someone they know has contracted COVID-19 or has come into close contact with someone who has COVID-19 [2]. Users are required to provide consent before interacting with the tool and are notified that: “The purpose of the Coronavirus Self-Checker is to help you make decisions about seeking appropriate medical care. This system is not intended for the diagnosis or treatment of disease, including COVID-19.” Prior to answering any questions, users are also encouraged to immediately call 911 if they are experiencing any life-threatening symptoms [2].

Users interact with this online, mobile-friendly tool in a short “conversation,” and answer questions about symptoms, exposure, underlying medical conditions, vaccination status, test results, and similar. Based on user responses and current CDC guidance about COVID-19, the Self-Checker recommends actions to seek appropriate levels of care and helpful resources [2]. For example, people who reported severe symptoms were recommended by the Self-Checker to seek care immediately, while people who reported mild symptoms were advised to self-monitor and isolate at home. On March 28, 2020, CDC made the decision tree, including the clinical and public health content for the Self-Checker, open source. This decision was taken to help state-, local-, and facility-level organizations and organizations in the digital health/technology space launch similar platforms.

Studies have documented that patients commonly seek health information online [4]. However, the existing literature on symptom checkers is mostly limited to small validation studies [5-7]. To date, no study has documented the implementation and use of a symptom checker in a large, governmental response to a global pandemic. The primary intention of the Self-Checker was to help reduce the burden on overstretched healthcare systems. Quantifying the number of recommendations delivered to users and the primary call-to-action (eg, themes) of those recommendations is important for understanding the tool’s potential impact. Therefore, the purpose of this study was to classify and describe recommendations delivered by the Self-Checker into themes and to analyze trends in conversations and usage of the tool over time.

## Methods

### Data Collection

Of the 20,276,748 conversations captured by the Self-Checker database between March 23, 2020, and April 19, 2021, in the United States, 16,718,667 (82.45%) completed conversations that ended with the delivery of a care message were analyzed. Conversations excluded from the analysis included (1) 411,332 conversations that were missing data for care messages and other important conversation variables; (2) 2,797,042 conversations for international users who were not the intended audience for the Self-Checker (the Self-Checker was designed for patients in the United States and recommendations were based on US CDC guidance); (3) 332,101 conversations for users who were in the 10-18 year age group and could not be categorized as adult or pediatric; and (4) 17,606 conversations that delivered care messages out of order (n=11,113) or delivered advice to US users that was meant for international users (to check with their Ministry of Health or local health department for location-specific guidance about COVID-19) (n=6493).

### Variables and Classification

Multiple variables were used to determine the self-reported health status of Self-Checker users. Because the Self-Checker uses the terms “ill” and “sick” interchangeably, in this analysis, we refer to an individual as “sick” or “not sick.” Two “yes or no” questions asked users if they felt sick or were caring for someone who felt sick: “Are you ill, or caring for someone who is ill?” and “Are you (they) feeling sick?” (Table 1). Current symptoms experienced, including life-threatening symptoms and underlying conditions, were also collected (see Tables A1 and A2 in Multimedia Appendix 1; timelines of Self-Checker updates to symptoms and underlying conditions lists are documented in Table A4 of Multimedia Appendix 1). Data capturing users’ responses about experiencing severe symptoms that required immediate medical attention were consolidated into a single life-threatening symptoms variable. Depending on how the decision-tree logic was adjusted during the study period, individuals could have responded to having life-threatening symptoms before or after answering a question about feeling sick. Individuals who responded “yes” to feeling sick could have experienced unspecified “other symptoms,” which were not associated with COVID-19. Location data were collected from two questions (ie, “Are you in the United States or a US territory right now?” and “Where in the United States or in which US territory are you currently located?”), and are described by census region or large city groups. Not all users shared state- or territory-level data that could categorize them into a census region. Lastly, depending on the age group selected, conversations were categorized as pediatric (eg, <2, 2-4, 5-9, 10-12, 13-17, <18 years old) or adult (eg, 18-29, 30-39, 40-49, 50-59, 60-64, 65-69, 70-79, 80+ years old). Age group options presented by the Self-Checker changed during the period of analysis as the tool evolved. The “<18 years old” age group was not presented to Self-Checker users concurrently with other pediatric age groups.

This analysis assessed several patient characteristics and outcomes. These include geographic location, age group, gender, person for whom the user was interacting with the Self-Checker, self-reported health status, and care messages delivered at the end of each conversation. Based on previous responses, the decision tree determined if a user would be presented with a specific question or delivered a care message at each step of the user journey. As a result, denominators for key variables vary.

Based on user responses, one of 18 different care messages was delivered at the end of each conversation with the Self-Checker (see Table A3 in Multimedia Appendix 1). For the purposes of this analysis, we manually classified conversations resulting in these care messages into three overarching themes: (1) seek care immediately; (2) take no action, or stay home and self-monitor for new or worsening symptoms (“take no action, or stay home and self-monitor”); or (3) conversation redirected. Theme-based consolidation of conversations was conducted to highlight the primary call-to-action indicated in the language of the care message delivered. Conversations classified within the “seek care immediately” theme delivered a care message that recommended sick individuals to visit an emergency room or urgent care, or to call a medical provider, telemedicine provider, or clinician advice line, based on the severity of their symptoms. Individuals who received these care messages: (1) indicated having life-threatening symptoms or a possible medical emergency, (2) had a medical condition that put them at risk of becoming more seriously ill, or (3) were in close contact or exposed to someone with COVID-19 in a healthcare or caregiving setting (eg, care center, nursing home, or homeless shelter) (Table A3 of Multimedia Appendix 1). Conversations classified within the “take no action, or stay home and self-monitor” theme delivered a care message that recommended individuals to take COVID-19 precautions, or for individuals experiencing mild or no symptoms to monitor for worsening or new symptoms of COVID-19 that would warrant contacting a medical provider. Individuals would have also received these care messages if they indicated not feeling sick. We classified these conversations within the same theme because the corresponding care messages were most likely to avert an unnecessary healthcare encounter, which was the original intent of the Self-Checker. Individuals who did not consent to using the Self-Checker or did not make any selections after starting a conversation received a care message to restart the tool and provide consent. These conversations were classified under the “conversation redirected” theme. Individuals who were too young to use the Self-Checker at the time of their conversation were redirected to other resources and also classified as “conversation redirected.”

The Self-Checker decision tree, including all care message recommendations, evolved over time; these were extensively reviewed by medical and public health professionals to ensure alignment with CDC guidance. Dates corresponding to each iteration of different care messages are documented in Table A3 of Multimedia Appendix 1.

The content and classification of one care message, Message 3, changed as the Self-Checker and CDC guidance about COVID-19 evolved (Table A3 of Multimedia Appendix 1). In early iterations, Message 3 advised users to call a healthcare provider for children under the age of 2 years who may have been sick, had contact with someone with COVID-19, or had recently been to an area where COVID-19 was spreading. We classified these conversations under the “seek care immediately” theme. Later iterations of Message 3 redirected age-ineligible users to visit CDC’s Coronavirus homepage to learn more about COVID-19 symptoms. These conversations were classified as “conversation redirected” until their discontinuation in September 2020.

Seven-day daily averages of Self-Checker conversations were plotted alongside 7-day daily averages of COVID-19 case counts from CDC’s COVID-19 Data Tracker [8] to illustrate use of the tool in the context of the pandemic. We analyzed trends in advice given by the Self-Checker by plotting the number of conversations per conversation theme with COVID-19 case counts. Information related to development (eg, guidance-based updates, new version releases) and marketing milestones (eg, language updates and launch of social media campaigns) that could have affected user engagement were obtained from the Self-Checker development team.

Data cleaning, analysis, and visualization were performed using SAS 9.4 and Microsoft Excel.

**Table 1:** User conversation characteristics (N=16,718,667).

Characteristic				Conversations, n (%)
**Self-reported health status**				
	Not sick			7,475,209 (44.7)
	Sick^a^			8,835,942 (52.9)
	Sick with life-threatening symptoms^b^			1,987,037 (22.5)
	Frequency missing^c^			407,516 (2.4)
**Gender**				
	Female			6,973,105 (64.0)
	Male			3,831,146 (35.2)
	I prefer not to say			86,481 (0.8)
	I don't know			4,089 (0.0)
	Frequency missing^d^			5,823,846 (34.8)
**Age (years)**				
	**Pediatric ages**			
		<2	46,186 (0.4)	
		2-4	109,877 (0.9)	
		5-9	168,575 (1.3)	
		10-12	105,271 (0.8)	
		13-17	395,064 (3.1)	
		<18^e^	221,834 (1.7)	
	**Adult ages**			
		18-29	3,146,922 (24.6)	
		30-39	2,891,699 (22.6)	
		40-49	2,259,745 (17.7)	
		50-59	1,770,067 (13.9)	
		60-64	680,892 (5.3)	
		65-69	466,333 (3.7)	
		70-79	413,406 (3.2)	
		80+	106,664 (0.8)	
	Frequency missing^f^			3,936,132 (23.5)
**Location^g^ by census region**				
	Island Regions			15,557 (0.1)
	Chicago, Los Angeles, and New York City			615,033 (3.8)
	Midwest			3,306,544 (20.6)
	Northeast (includes PR^h^)			2,787,362 (17.4)
	South			5,827,250 (36.4)
	West			3,467,348 (21.7)
	Frequency missing^i^			699,573 (4.2)
**Responding for**				
	Myself only			10,813,620 (83.2)
	Someone else			2,178,535 (16.8)
	Frequency missing^j^			3,726,512 (22.3)

^a^Data about users feeling sick were collected in two questions: “Are you ill, or caring for someone who is ill?” and “Are you (they) feeling sick?”.

^b^22.49% of conversations were initiated for individuals who experienced life-threatening symptoms. This percentage represents 1,987,037 among 8,835,942 conversations for individuals who reported feeling sick, but accounts for only 11.89% (1,987,037/16,718,667) of all conversations that were initiated with the Self-Checker during the study period.

^c^Data about users feeling sick (“Are you ill, or caring for someone who is ill?” or “Are you [they] feeling sick?”) were not captured for 2.44% (n=407,516) of conversations. Outcome messages were still delivered at the end of these conversations: 12.77% of conversations resulted in recommendations to seek care immediately; 15.89% resulted in recommendations to take no action, or stay home and self-monitor; and 71.34% of conversations redirected users out of the Self-Checker to other resources.

^d^Gender data were not captured for 34.83% (n=5,823,846) of conversations because users did not receive the question about gender (“What is your [their] gender?”) ahead of being triaged out of the Self-Checker due to ineligibility or for having reported life-threatening symptoms.

^e^Different age groups were introduced to the Self-Checker during the analysis period. At one point, “<18 years old” was a valid option to select from for users answering for an individual who fell within the pediatric group. At other times, the “<18 years old” age group was replaced by more granular age groups.

^f^Age data were not captured for 23.54% (n=3,936,132) of conversations because users did not receive the question about age (“What is your [their] age?”) ahead of being triaged out of the Self-Checker due to ineligibility or for having reported life-threatening symptoms.

^g^High-level location data were collected for all users of the Self-Checker (“Are you in or outside of the United States?”). However, users were not required to provide state-level location data or information about their specific census region (census regions are defined as: Island Regions=Guam, American Samoa, Marshall Islands, Northern Mariana Islands, US Virgin Islands; Midwest=Illinois, Indiana, Iowa, Kansas, Michigan, Minnesota, Missouri, Nebraska, North Dakota, Ohio, South Dakota, Wisconsin; Northeast=Connecticut, Maine, Massachusetts, New Hampshire, New Jersey, New York, Pennsylvania, Puerto Rico, Rhode Island, Vermont; South=Alabama, Arkansas, Delaware, District of Columbia, Florida, Georgia, Kentucky, Louisiana, Maryland, Mississippi, North Carolina, Oklahoma, South Carolina, Tennessee, Texas, Virginia, West Virginia; and West=Alaska, Arizona, California, Colorado, Hawaii, Idaho, Oregon, Montana, New Mexico, Nevada, Utah, Washington, Wyoming.)

^h^PR: Puerto Rico.

^i^4.18% (n=699,573) of users could not be categorized into a census region because they did not provide state-level location information when asked “Where in the United States are you located?” or “Where in the United States or in which US territory are you currently located?”.

^j^“Who for” data (“Are you answering for yourself or someone else?”) were not captured for 22.29% (n=3,726,512) of conversations because users did not receive the question ahead of being triaged out of the Self-Checker due to ineligibility or for having reported life-threatening symptoms.

### Ethical Considerations

All users who engaged with the Self-Checker were asked to consent to using the tool before providing personal information for themselves or someone else, and before receiving advice from the tool. This activity was reviewed by CDC and was conducted consistent with applicable federal law and CDC policy (45 C.F.R. part 46; 21 C.F.R. part 56; 42 U.S.C. Sect. 241(d); 5 U.S.C. Sect. 552a; 44 U.S.C. Sect. 3501 et seq).

## Results

### Overall Demographics

Between March 23, 2020, and April 19, 2021, a total of 16,718,667 completed conversations between US users and the Self-Checker were analyzed. As shown in Table 1, data for key variables were not collected for all conversations and denominators vary; however, 83.23% (10,813,620/12,992,155) of conversations were initiated for individuals who were seeking COVID-19 care guidance for themselves as opposed to for someone else. Over half of the conversations were initiated for people who were sick (n=8,835,942, 52.85%); among them, 22.49% (1,987,037/8,835,942) reported experiencing life-threatening symptoms (Table A1 of Multimedia Appendix 1). As shown in Table 1, the majority of conversations analyzed were for females (6,973,105/10,894,821, 64.00%) and adults 18 years and older (11,735,728/12,782,535, 91.81%). Counts in the number of conversations were lower with older age, with the most conversations occurring among 18-29–year-olds (n=3,146,922, 24.62%) and the least number of conversations occurring for individuals 80 years and older (n=106,664, 0.83%) (Table 1). Users who shared location information (16,019,094/16,718,667, 95.82%) were from the following census regions: South (36.4%), West (21.7%), Midwest (20.6%), Northeast (17.4%), Island regions (0.1%), and less than 4% were located in 3 large cities (Chicago, Los Angeles, and New York City) (Table 1).

### Distribution by Theme for All Conversations

The majority of conversations (11,580,738/16,718,667, 69.27%) ended with recommendations to take no action, or stay home and self-monitor (Table A3 of Multimedia Appendix 1). Less than a third of conversations (n=4,822,138, 28.84%) advised users to seek immediate care, and only 1.89% (n=315,791) of conversations redirected users out of the Self-Checker without receiving a care recommendation (Table A3 of Multimedia Appendix 1). The data show that 50.56% (n=5,854,902) of conversations classified under the “take no action, or stay home and self-monitor” theme were initiated for individuals who were not sick (received Message 1). All other conversations classified within this theme occurred for individuals who reported feeling sick but reported mild symptoms that may have been unrelated to COVID-19, or may have had close contact with someone with COVID-19. Those who had close contact with someone with COVID-19 were recommended to contact a provider if COVID-19 symptoms developed or worsened (Table A3 of Multimedia Appendix 1).

### Distribution by Theme for Not Sick and Sick People

Table 2 shows the distribution of conversations for not sick and sick individuals (with and without life-threatening symptoms) across conversation themes. Percentages by self-reported health status and among all conversations are displayed. Less than 1% (0.17%) of conversations initiated for individuals who reported not being sick resulted in a recommendation to “seek care immediately,” as did 40.45% of conversations for sick individuals without life-threatening symptoms and over 99.98% of conversations for sick individuals with life-threatening symptoms. Overall, 29.24% of all Self-Checker conversations advised to seek immediate care (Table 2).

The majority of conversations initiated for individuals who were not sick (7,460,553/7,475,209, 99.80%) resulted in advice to “take no action, or stay home and self-monitor.” As indicated in Table 2, these conversations represented 45.74% of all Self-Checker conversations during the analysis period. Among 6,848,905 conversations initiated for sick individuals without life-threatening symptoms, 59.21% (n=4,054,966) resulted in a recommendation to “take no action, or stay home and self-monitor” (Table 2; see Table A1 in Multimedia Appendix 1 for a list of life-threatening symptoms). This group of conversations represents 24.86% of all conversations initiated with the Self-Checker that had a self-reported health status recorded (n=16,311,151), and occurred for individuals who (1) reported symptoms that may have been unrelated to COVID-19; (2) reported feeling sick but did not report any symptoms; or (3) were aged 19 to 64 years and reported symptoms other than cough, fever, or mild or moderate difficulty breathing. The remaining 0.02% (n=462) of conversations for sick individuals with life-threatening symptoms resulted in this same recommendation, which varied from the intended pathway determined by CDC guidance based on reported symptoms. In total, 70.6% of all Self-Checker conversations resulted in a recommendation to “take no action, or stay home and self-monitor.” The majority of redirected conversations were among sick individuals without life-threatening symptoms (Table 2).

**Table 2:** Classification of conversations by self-reported health status and conversation theme (N=16,311,151).^a^

Conversation theme	Not sick (n=7,475,209), n (% of theme), (% of all conversations)	Sick, n (% of theme), (% of all conversations)		Total conversations, n (%)
		Without life-threatening symptoms (n=6,848,905)	With life-threatening symptoms (n=1,987,037)	
Seek care immediately	13,048^b^ (0.17), (0.08)	2,770,489^c^ (40.45), (16.99)	1,986,575 (99.98), (12.18)	4,770,112 (29.24)
Take no action, or stay home and self-monitor	7,460,553 (99.8), (45.74)	4,054,966^d^ (59.21), (24.86)	461^e^ (0.02), (0.00)	11,515,980 (70.60)
Conversation redirected	1608 (0.02), (0.01)	23,450 (0.34), (0.14)	1 (0.00), (0.00)	25,059 (0.15)

^a^This table displays a reduced sample of 16,311,151 conversations because self-reported health status (“Are you ill, or caring for someone who is ill?” or “Are you [they] feeling sick?”) was not collected for 407,516 conversations ahead of users being triaged out of the Self-Checker due to ineligibility or reporting life-threatening symptoms.

^b^13,048 individuals who reported not feeling sick but were recommended by the Self-Checker to “seek care immediately” lived in a care center, nursing home, or homeless shelter where they may have been in close contact with someone who may have COVID-19. The Self-Checker delivered Care Message 25 to these individuals.

^c^Sick individuals without life-threatening symptoms who were recommended to “seek care immediately” were described as: (1) having a medical emergency, (2) children <2 years old who could be sick, (3) people under age 19 or ≥65 years with a comorbidity, (4) people who lived in a group or congregate care setting, (5) people 65 years and older with at least 2 COVID-19 symptoms, (6) adults aged 19-64 years with one COVID-19 symptom and who worked/volunteered in a congregate care setting and had close contact with someone with COVID-19, or (7) children with at least one COVID-19 symptom.

^d^Sick individuals without life-threatening symptoms who were recommended to “take no action, or stay home and self-monitor” were described as: (1) having no specific or any symptom of COVID-19; or (2) age 19 to 64 years, who only experienced one secondary symptom related to COVID-19 (excluding mild or moderate difficulty breathing).

^e^461 conversations conducted between March 30, 2020, and April 27, 2020, when the Self-Checker was repeatedly adjusting to rapid changes in Centers for Disease Control and Prevention guidance, were for individuals who received Care Message 8 or Care Message 10 (see Table A3 of Multimedia Appendix 1). All individuals reported one symptom of “ribs (were) pulling in with each breath (retractions).”

### Trends in Overall Self-Checker Usage

**Figure 1:**
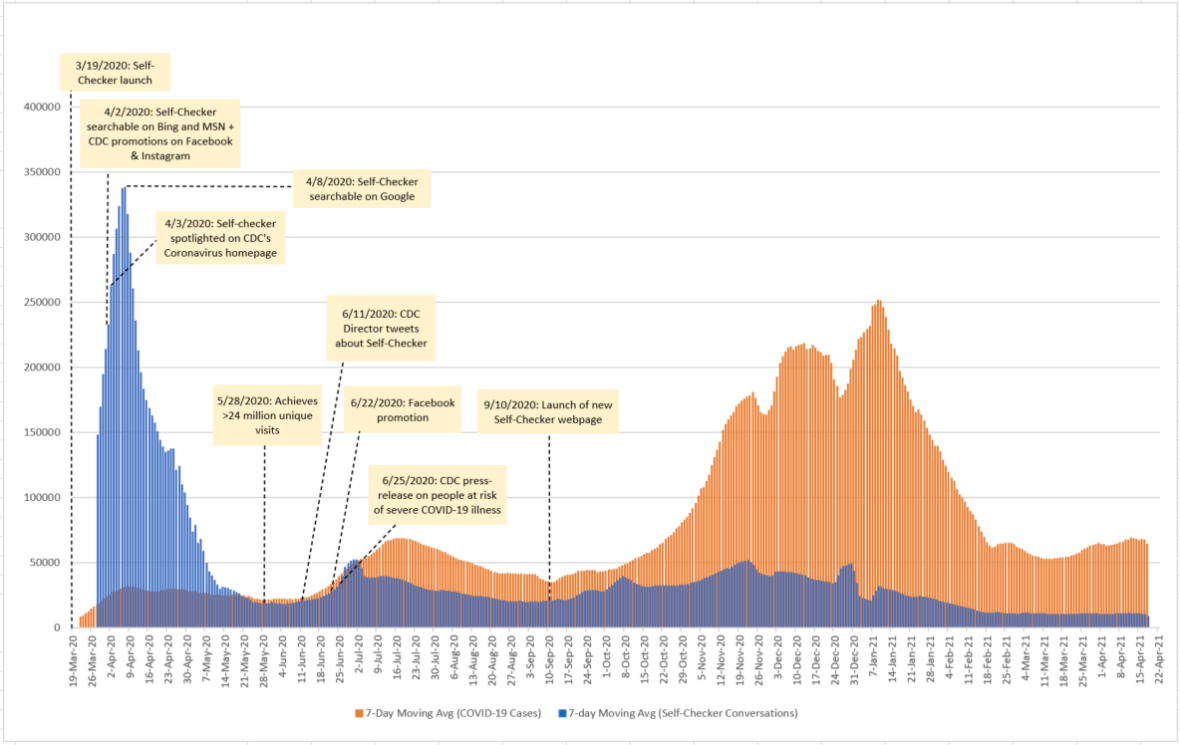
Trends in 7-day moving averages of COVID-19 case counts and Self-Checker conversations in the United States between March 23, 2020, and April 19, 2021, highlighting marketing milestones that may have influenced user engagement with the tool during the analysis period. CDC: Centers for Disease Control and Prevention

As shown in Figure 1, following the launch of the Self-Checker in mid-March 2020, the 7-day rolling average of conversations peaked on April 8, 2020, at 338,551 conversations. Thereafter, counts of conversations fell by over 90% (to 23,933 conversations) and remained at lower levels from late-May 2020 onward. Occasional, smaller increases in conversations often preceded large increases in COVID-19 case counts. For example, between September 7, 2020, and November 23, 2020, engagement with the tool increased by 68% as COVID-19 cases increased by approximately 78% within the same time frame. Another peak in conversations occurred in December 2020, which preceded a rise in COVID-19 cases in January 2021. By mid-February 2021, conversations were at their lowest numbers during the investigation period, similar to COVID-19 case counts (Figure 1).

### Trends in Self-Checker Conversations by Conversation Theme

**Figure 2:**
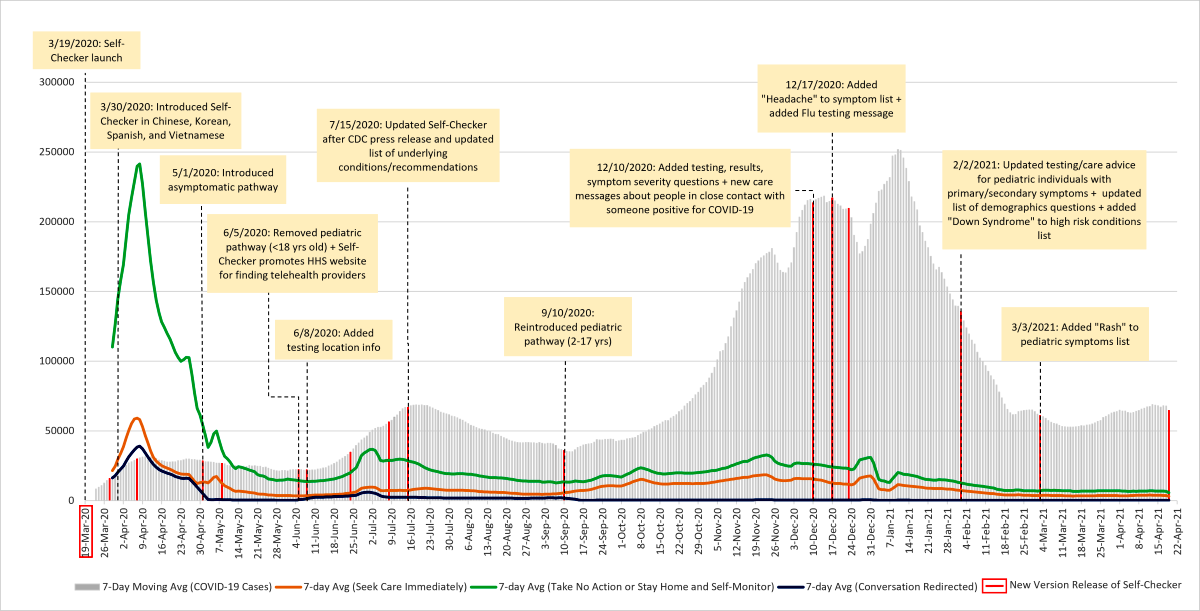
Trends in the 7-day moving averages of COVID-19 case counts and Self-Checker conversations by overarching conversation theme in the United States between March 23, 2020, and April 19, 2021, highlighting development milestones and chronicles updates to the Self-Checker, as shown by the red bars. CDC: Centers for Disease Control and Prevention. HHS: U.S. Department of Health and Human Services.

Figure 2 illustrates trends in the 7-day rolling averages of conversations per theme and COVID-19 case counts. Overall, conversations recommending to “take no action, or stay home and self-monitor” occurred consistently more often than conversations recommending the user to “seek care immediately.” During the April 2020 peak, the number of conversations that recommended to “take no action, or stay home and self-monitor” (241,426 conversations) was four times higher than the number of conversations that recommended to “seek care immediately” (58,014 conversations). Similarly, as Self-Checker engagement rose in mid-June and peaked on July 2, 2020, conversations classified under the “take no action, or stay home and self-monitor” theme occurred four times more frequently than conversations in the “seek care immediately” theme (36,790 vs 9689 conversations, respectively). This trend continued between December 24, 2020, and January 11, 2021, with conversations recommending to “take no action, or stay home and self-monitor” occurring twice as often as conversations recommending to “seek care immediately.” Thereafter, even as Self-Checker engagement steadily declined, conversations recommending users to “take no action, or stay home and self-monitor” continued to occur nearly twice as often as conversations classified under the “seek care immediately” theme (average of 6900 vs 3600 conversations in the last 50 days of the study period, respectively).

## Discussion

### Principal Findings

This analysis highlights characteristics of over 16 million completed Self-Checker conversations. Our findings demonstrate three important points about users and conversations initiated with the Self-Checker: (1) during the pandemic, individuals who did not need immediate medical care engaged with the tool more often than those who required immediate care; (2) high proportions of conversations initiated for sick (n=4,055,427, 46%) and not sick (n=7,460,553, 99.8%) individuals, based on the symptoms reported, did not end with recommendations to seek immediate care, either in-person or via a remote modality such as telehealth (Table 2); and (3) if all users followed Self-Checker recommendations, it is possible that 69.3% of them would not have pursued in-person or remote medical care, potentially decreasing the burden on the US healthcare system, as indicated in Table A3 of Multimedia Appendix 1.

A large proportion of conversations initiated by individuals who were not sick recommended to “take no action or stay home and self-monitor.” Half of these conversations recommended Message 1 (“Sounds like you are feeling okay”), as users reported no symptoms (Table A3 of Multimedia Appendix 1). These findings suggest that most individuals who engaged with the tool and did not require immediate or any medical attention were potentially only seeking information about COVID-19 online.

One report of an online triage tool that focused on providing appropriate COVID-19 testing recommendations to users in Switzerland indicated that nearly 70% of users would have contacted healthcare systems in the absence of the tool [9]. Galmiche et al [10] further reported a marked reduction in the ratio of emergency center calls to hospitalizations after the implementation of a similar system in France during the COVID-19 pandemic. Both studies suggest a possible impact of automated symptom-checking tools on accurately directing patients to the most appropriate care, particularly when in-person or remote medical care is not urgently needed.

Evaluations of similar triage tools that compare tool-provided recommendations to caregiver assessments have found that automated symptom-checking tools tend to advise patients to seek higher levels of care than necessary [5,11,12]. For example, Hill et al [5] found that among 688 standardized patient vignettes, 40% of vignettes in the nonurgent and self-care category advised to seek urgent or emergent care. Price et al [12] evaluated a clinical algorithm designed to help parents and adult caregivers determine if a child’s influenza-like illness required emergency care. To avoid misclassification of high-risk cases and in consideration of safety concerns, the algorithm regarded 87.4% of patients as “high risk” and may have overtriaged patients to the emergency department. To identify the most appropriate criteria for delivering needed and safe advice to users, Price et al [12] suggested examining a wider range of clinical questions to achieve higher specificity of triage advice without forgoing patient safety. We were unable to evaluate if the advice delivered by the Self-Checker to “seek care immediately” followed this trend of conservative caution, but it is clear that the tool delivered this recommendation to users who reported having: (1) life-threatening symptoms or a possible medical emergency, (2) a medical condition that put them at risk of becoming more seriously ill, or (3) close contact or exposure to someone with COVID-19 in a healthcare or caregiving setting. The CDC Self-Checker reserved this recommendation for people who reported the most severe illness or highest vulnerability to becoming sick with COVID-19. In addition to demographic and clinical questions, users were asked a variety of nonclinical questions to better understand their circumstances for engaging with the tool (eg, questions about close contacts, residence in a long-term care setting, working in a healthcare setting, wearing personal protective equipment). By understanding the full experience of each individual, the advice from the Self-Checker could be better targeted. Ultimately, we observed that the Self-Checker delivered recommendations to “take no action, or stay home and self-monitor” more often than to “seek care immediately.”

For a brief period following its launch, the Self-Checker provided advice to some users that did not fully match their responses. Given the rapid development of the tool during an emergency response, we observed only 0.003% of all conversations where the algorithm varied from the intended pathway. Overall, recommendations from the Self-Checker were highly consistent with CDC guidelines, with over 99.9% of recommendations being delivered in accordance with the intended pathway.

Table A3 of Multimedia Appendix 1 illustrates that 1.89% of Self-Checker users were redirected out of the Self-Checker to other resources. Because repeated conversations from the same device or location were not linked in the Self-Checker, it was impossible to determine if redirected, age-eligible users (ie, users who did not consent or make a selection after beginning a conversation) re-engaged with the tool. It is also unclear how frequently the Self-Checker would have recommended repeat users to “seek care immediately” or “take no action, or stay home and self-monitor.”

We observed substantially higher use of the Self-Checker in the first months after its launch, as illustrated in Figure 1. Several factors may explain this early surge in use. Following the launch of the Self-Checker in March 2020, online promotions of the tool by CDC and others were ongoing and could have impacted user engagement. The first such promotional events occurred when the tool was released in four non-English languages (simplified Chinese, Vietnamese, Spanish, and Korean), making it accessible to non-English speakers in March 2020 (Figure 1). The Self-Checker then became searchable on MSN, Bing, and Google by the end of the first week of April 2020. CDC also promoted the tool on social media and the CDC Coronavirus homepage (Figure 1). These combined efforts may have increased conversations to the highest levels observed during the study period.

Additional promotional events in June 2020 (eg, CDC Director tweets, CDC Facebook promotion, updates to CDC’s underlying conditions list shared in a press release) may have resulted in a substantial increase in overall conversations (by 155% from 20,423 to 52,169 conversations) between June 12, 2020, and July 1, 2020 (Figure 1). Conversations classified within the “take no action, or stay home and self-monitor” and “seek care immediately” themes increased by 161% and 142%, respectively (Figure 2). We consistently observed that trends in COVID-19 case counts followed trends in engagement with the Self-Checker during this period in the pandemic. These patterns in conversations and case counts could be attributed to promotions of the tool and also to the expected lag of COVID-19 case reporting by state and local health departments.

Fluctuations in engagement with the Self-Checker may have also been attributed to revisions to CDC guidance about COVID-19, which were reflected in new releases of the tool (Figure 2, Table A4 of Multimedia Appendix 1). One example is when Self-Checker’s pediatric pathway for 2-17–year-olds was discontinued on June 5, 2020, due to changes to official CDC guidance about COVID-19 in the pediatric population. Prior to the pediatric pathway being reintroduced on September 10, 2020, all conversations for individuals aged 2-17 years (218,873 conversations) were redirected out of the Self-Checker to the CDC website. In the 3 months following reintroduction of the pediatric pathway, the 7-day rolling average of conversations within the pediatric group more than doubled among the “seek care immediately” and “take no action, or stay home and self-monitor” themes. Other fluctuations in engagement with the Self-Checker may have been attributed to CDC making its decision tree open source and available to the public and other organizations for use.

Further analyses that focus on user intentions to follow recommendations and user satisfaction of the recommendation received are planned to measure the impact of Self-Checker advice delivered to care-seeking individuals and its applicability during future public health emergencies.

### Limitations

There are some limitations to this investigation. Approximately 17.5% of conversations were excluded from the analysis for at least one of the following reasons: (1) users could not be categorized as adult or pediatric, (2) conversations did not result in the delivery of a care message, (3) conversations occurred with users located outside of the continental United States or territories, or (4) conversations delivered care messages outside of the scope of our analysis. The earliest versions of the Self-Checker were being updated repeatedly to reflect rapidly changing CDC guidance about COVID-19. Therefore, these rapid adjustments to the tool may have resulted in data capture challenges that surfaced within our analytic data set.

Due to missing data and an inability to identify the number of unique users who interacted with the Self-Checker, the data presented in this manuscript may not be accurately representative of the individuals who engaged in conversations. User demographics, information for whom the user was engaging with the Self-Checker for, and self-reported health status data were missing for up to 35% of conversations. These missing data points were a consequence of users being triaged out of the tool before encountering questions for key variables due to ineligibility or reporting life-threatening symptoms. As location was an optional field that was not introduced to the Self-Checker until May 2020, location data are missing for a fraction of conversations. In addition, because all conversations with the Self-Checker are assigned a unique identifier (“conversation ID”) once started, it is possible that users started multiple conversations with the Self-Checker during the period of analysis.

Furthermore, the Self-Checker, being a web-based tool, is only accessible to individuals who are digitally literate and have access to the internet or smartphones. As a result, vulnerable populations—including those who are unfamiliar or uncomfortable with using digital platforms, such as the elderly and people living in underprivileged communities, who may be experiencing the greatest impacts of COVID-19 compared to others—may be unable to use the tool.

In an effort to allow public health agencies and healthcare partners to adopt and provide recommendations to the communities they serve based on CDC’s most current COVID-19 guidance, CDC made the decision tree for the Self-Checker open source and publicly available. However, it has not been possible to track if public users have deployed and are maintaining exact or modified versions of the Self-Checker on their own websites. If external users have launched their own symptom-checking tools based on the Self-Checker, we are not aware of the levels of user engagement with those tools.

Lastly, we could not determine if users followed the advice delivered by the Self-Checker and could not confirm whether individuals who did seek care took advantage of remote care modalities such as telehealth, instead of seeking in-person care.

### Perspectives for Future Research

This study highlights the usefulness of the Self-Checker for guiding users to the most appropriate level of healthcare; it encourages opportunities for care modalities such as telehealth to alleviate the burden on healthcare systems. Furthermore, given that over 85% of people in the United States have smartphones, web-based tools such as the Self-Checker could potentially expand the reach of medical care to underserved populations during public health emergencies [13]. Based on our findings, we offer four main lessons learned. First, future updates of the tool could include more questions that allow for better understanding of user characteristics. Initially, the tool did not collect detailed user characteristics, as early assessments showed user hesitation for providing individual-level details. As the pandemic progressed and new information became available, the Self-Checker expanded to capture more demographic data on users (eg, race, ethnicity, gender assignment at birth, optional location information at the zip-code level). Analyzing these data points could help identify vulnerable populations impacted by the pandemic and location-specific trends.

Second, asking users about their understanding of the advice received, their course of action following engagement with the Self-Checker, and their satisfaction with the tool may provide additional data to inform the public health response and further assess the utility of such platforms. The latest version of the tool, introduced after this analysis was conducted, includes one Yes-No question (“Was this screening tool helpful?”) and one Likert-scale question (“Based on the information provided [by the Self-Checker], how likely are you to follow these recommendations?”) to capture user experience and intention to follow recommendations. More detailed inquiries about if the user had interacted with the Self-Checker in the past and what care modality users already pursued or will pursue following their current interaction could be explored.

Third, it is imperative that automated tools like the Self-Checker are thoroughly tested to ensure that advice delivered to users aligns with evidence-based guidance, even when guidance is quickly growing more complex. In the midst of a global pandemic, strategies to prevent errors in decision-tree logic (eg, manual or automated data quality processes, including code-based consistency checks) and testing resources should be available to ensure that particularly sick individuals are delivered appropriate care advice based on their symptoms.

Lastly, in an effort to rapidly share evolving clinical and public health information in a coordinated way, CDC could make decision trees that help navigate through CDC guidance, such as that used to power the Self-Checker, open source. Making content for web-based tools like the Self-Checker open source allows for public health agencies, healthcare partners, and other stakeholders to adopt rapidly changing CDC guidance and integrate public health recommendations for action into health-tech systems.

These lessons learned are aimed at understanding the impact of the Self-Checker on freeing up healthcare resources and relieving provider burden by recommending patients with noncritical care needs to manage symptoms at home; this also offers safer options to providers and patients by reducing infectious exposures during emergency situations. The ability of the Self-Checker to facilitate patient-driven medical decision-making may assist in the response to similar public health emergencies in the future.

## Appendix

Multimedia Appendix 1 Supplementary data; Tables A1-A4.

## Abbreviations

CDC: Centers for Disease Control and Prevention
