# Supplementary material for: Engagement With the Centers for Disease Control and Prevention Coronavirus Self-Checker and Guidance Provided to Users in the United States From March 23, 2020, to April 19, 2021: Thematic and Trend Analysis
Source: J Med Internet Res. 2023 Mar 10;25:e39054. doi: 10.2196/39054 (PMC10039408; doi:10.2196/39054)
Supplement: Multimedia Appendix 1 [file jmir_v25i1e39054_app1.docx]

Table A1. List of Life-Threatening Symptoms Presented by the Self-Checker, March 23, 2020 - April 19, 2021^a^

| **Life-threatening symptoms** |
| --- |
| Coughing up blood (more than about 1 teaspoon) |
| Dehydration (dry lips and mouth, not urinating much, sunken eyes) |
| Extreme difficulty breathing (such as gasping for air, being unable to walk or talk without catching your (their) breath, severe wheezing, nostrils flaring, grunting, or using extra muscles around the chest to help breathe) |
| Extremely fast or shallow breathing |
| Frequent vomiting |
| Mild: you can get enough air in your lungs but your chest feels tight when you take a deep breath |
| Moderate to severe difficulty breathing (unable to speak full sentences) |
| New serious disorientation (acting confused) |
| Pale, gray, or blue-colored skin, lips, or nail beds, depending on skin tone |
| Refusing to drink liquids |
| Ribs are pulling in with each breath (retractions) |
| New seizure or seizures that won’t stop |
| Not waking up or not interacting when awake |
| Severe and constant dizziness or lightheadedness |
| Severe and constant pain or pressure in the chest |
| Severe: gasping for air or cannot talk without catching your breath |
| Signs of low blood pressure (too weak to stand, light-headed, feeling cold, pale, clammy skin, light-headed, too weak to stand) |
| Slurred speech or difficulty speaking (new or worsening) |
| So irritable that the child does not want to be held |
| Unconscious or very difficult to wake up |

^a^ The list of life-threatening symptoms varied by age and was revised multiple times during the study period in accordance with changing CDC guidance. The list is condensed to exclude variations in the language used to describe life-threatening symptoms presented to users at different times during the study period.

Table A2. List of Underlying Conditions Presented by the Self-Checker, March 23, 2020 - April 19, 2021^a^

| **Underlying Conditions/Comorbidities** |
| --- |
| Blood disorder, such as sickle cell disease or thalassemia |
| Born premature |
| Cancer |
| Cerebrovascular disease or neurologic condition, such as stroke or dementia |
| Chronic kidney disease requiring hemodialysis |
| Chronic lung disease, moderate to severe asthma, or smoking |
| Chronic lung disease, such as moderate to severe asthma, COPD (chronic obstructive pulmonary disease), cystic fibrosis, or pulmonary fibrosis |
| Cirrhosis of the liver |
| Congestive heart failure |
| Diabetes, chronic kidney disease, or liver disease |
| Down syndrome or Down’s syndrome |
| High blood pressure |
| HIV |
| Neurologic condition, such as cerebral palsy |
| Pregnancy |
| Receiving chemotherapy or radiation for cancer |
| Serious heart condition, such as congenital heart defect |
| Serious heart condition, such as heart failure, cardiomyopathy, heart attack, or blocked arteries to the heart |
| Severe obesity (Body Mass Index [BMI] > 40) |
| Smoking or vaping |
| Weakened immune system (cancer treatment, prolonged use of steroids, transplant or HIV/AIDS) |
| Weakened immune system or taking medications that may cause immune suppression |

^a^ The list of underlying conditions varied by age and was revised multiple times during the study period in accordance with changing CDC guidance. The list is condensed to exclude variations in the language used to describe underlying conditions presented to users at different times during the study period.

Table A3. Counts of Conversations per Care Message and Conversation Theme (N=16,718,667)

| **Conversation Theme** | **Care Message (Message Number, Message Language)** | **Conversations Resulting in Each Care Message** n (% within theme) | **Conversations per Conversation Theme** n (% of total sample) |
| --- | --- | --- | --- |
| **Conversation Redirected^a^** | **MSG3**  This Coronavirus Self-Checker is for people who are at least 2 years old. Visit the CDC website to get information on COVID-19 and children. **(4/24/2020-6/4/2020)** This Coronavirus Self-Checker is for people who are at least 18 years old. Visit the CDC website to get information on COVID-19 and younger people. **(6/5/2020-9/9/2020)** | 226,421 (71.7) | 315,791 (1.9) |
|  | **MSG12** Please consent to use the Coronavirus Self-Checker. Refresh the page to start again. | 51,257 (10.1) |  |
|  | **MSG0** You have not made a selection. Please start again and select options for each question so that I can help give you advice. | 31,975 (0.2) |  |
|  | **MSG20** Please ask your parent or guardian to help you complete these questions. | 6,138 (1.9) |  |
| **Seek care immediately^b^** | **MSG5** Call your medical provider, clinician advice line, or telemedicine provider as soon as possible. Your symptoms may be related to COVID-19. You also have medical conditions that may put you at risk of becoming more seriously ill. | 1,616,451 (33.5) | 4,822,138 (28.8) |
|  | **MSG4** Based on your symptoms, you may need urgent medical care. Please call 911 or go to the nearest emergency department. | 1,209,210 (25.1) |  |
|  | **MSG9**  Stay home and take care of yourself in home isolation. Call a medical provider within 24 hours. You have one or more symptom(s) that may be related to COVID-19. Call your healthcare provider, clinician advice line, or telemedicine provider within 24 hours. Start home isolation.  This means stay home except to get medical care, and do not go to work, school, or public areas. Do not use public transportation or ride sharing. Be sure to get care if you feel worse or you think it is an emergency. **(3/19/2020-6/4/2020)**  Stay home and take care of yourself. Call a medical provider within 24 hours. Your symptom(s) may be related to COVID-19. Call your healthcare provider, clinician advice line, or telemedicine provider within 24 hours.   - Stay home except to get medical care. - Do not go to work, school, or public areas. - Do not use public transportation or ride sharing.   If you feel worse or think this is an emergency, seek medical care.  Find telehealth services **(6/5/2020- 9/9/2020)**  Stay home (keep them home) and take care of yourself (them). Call your (their) medical provider. Your (their) symptom(s) may be related to COVID-19. Call your (their) medical provider, clinician advice line, or telemedicine provider. Stay home (keep them home) except to get medical care.   - Do not go to work, school, or public areas including grocery stores, pharmacies, or restaurants. Consider delivery options for food and medicine. - Do not use public transportation or ride sharing.   If you (they) feel worse, and you think it is an emergency, call 911 or seek medical care immediately.  Consider using telehealth services if available. **(9/10/2020 – 12/9/2020)**  Stay home (keep them home) and take care of yourself (them). Call your (their) medical provider. Your (their) symptom(s) may be related to COVID-19. Call your (their) medical provider, clinician advice line, or telemedicine provider. Stay home (keep them home) except to get medical care.   - Do not go to work, school, or public areas including grocery stores, pharmacies, or restaurants. Consider delivery options for food and medicine. - Do not use public transportation or ride sharing. - Cover your coughs and sneezes with a tissue. - Wash your hands often with soap and water. - Avoid close contact with other people. Stay at least 6 feet away from other people. - Wear a mask when around others.   If you (they) feel worse, and you think it is an emergency, call 911 or seek medical care immediately.  Consider using telehealth services if available. **(12/10/2020 – 4/19/2021)** | 1,104,237 (22.9) |  |
|  | **MSG2** Call 911 - You may be having a medical emergency. Immediate medical attention is needed. Tell the 911 operator if you have been in contact with someone with COVID-19 or if you have recently been to an area where COVID-19 is spreading. **(3/28/2020-4/30/2020)** | 795,438 (16.5) |  |
|  | **MSG7** Contact a healthcare provider in the long-term care facility where you live. Tell a caregiver in your facility that you are sick and need to see a medical provider as soon as possible. Stay in your room as much as possible except to get medical care. **(3/19/2020-9/9/2020)** Contact a medical provider in the care center, nursing home, or homeless shelter where you (they) live. Tell a caregiver in your facility that you are sick and need to see a medical provider as soon as possible. Stay in your room as much as possible except to get medical care. **(9/10/2020-4/19/2021)** | 46,487 (1) |  |
|  | **MSG19** This tool is intended for people 2 years or older. Please call the child/s medical provider, clinician advice line, or telemedicine provider if your child is less than 2 years old and sick. | 22,437 (0.5) |  |
|  | **MSG3** Sorry, this Coronavirus Self-Checker is for people who are at least 2 years old.  Call your child’s healthcare provider today. Tell them if your sick child has had contact with someone with COVID-19 or if they have recently been to an area where COVID-19 is spreading. **(3/19/2020-4/23/2020)** | 14,830 (0.3) |  |
|  | **MSG25** Contact a medical provider in the care center, nursing home, or homeless shelter where you (they) live.  Tell a caregiver in your (their) facility that you (they) may have been in close contact with someone who may have COVID-19. They can help prevent infections at your (their) facility or shelter.  Help protect others from getting sick:   - Stay in your room as much as possible except to get medical care. - Cover your coughs and sneezes with a tissue. - Clean your hands often. - Avoid close contact with other people. Stay at least 6 feet away from other people. - Wear a mask when around others, if possible. - Monitor your health and notify a medical provider if you start to feel sick. | 13,048 (0.3) |  |
| **Take no action, or stay home and self-monitor^c^** | **MSG1** Sounds like you are feeling ok. | 5,854,902 (50.6) | 11,580,738 (69.3) |
|  | **MSG8**  Stay home except to get medical care and take care of yourself. Call your provider if you get worse. Sorry you are feeling ill. You have one or more symptom(s) that may be related to COVID-19. **(3/19/2020-4/30/2020)**  Stay home and take care of yourself. Call your provider if you get worse. Sorry you are feeling ill. You have one or more symptoms that may be related to COVID-19. Stay home and take care of yourself. This means to stay home except to get medical care, and do not go to work, school, or public areas. If you feel worse or you think it is an emergency, seek medical care. **(5/1/2020-7/12/2020)**  Stay home (keep them home) and take care of yourself (them). Call your (their) medical provider if you get worse. Sorry you are (or your child is) not feeling well. Your symptoms may be related to COVID-19. Stay home (keep them home) except to get medical care.   - Do not go to work, school, or public areas including grocery stores, pharmacies, or restaurants. Consider delivery options for food and medicine. - Do not use public transportation or ride sharing.   If you (they) feel worse, and you (they) think it is an emergency, call 911 or seek medical care immediately. **(7/13/2020-12/9/2020)**  Stay home (keep them home) and take care of yourself (them). Call your (their) medical provider if you get worse. Sorry you are (or your child is) not feeling well. Your symptoms may be related to COVID-19. Stay home (keep them home) except to get medical care.   - Do not go to work, school, or public areas including grocery stores, pharmacies, or restaurants. Consider delivery options for food and medicine. - Do not use public transportation or ride sharing. - Cover your coughs and sneezes with a tissue. - Wash your hands often with soap and water. - Avoid close contact with other people. Stay at least 6 feet away from other people. - Wear a mask when around others.   If you (they) feel worse, and you (they) think it is an emergency, call 911 or seek medical care immediately. **(12/10/2020-4/19/2021)** | 3,239,535 (28) |  |
|  | **MSG18** Stay home for 14 days from the day you last had contact. Watch for COVID-19 symptoms such as fever, cough, or difficulty breathing. If you develop any of these symptoms, call your medical provider, clinician advice line, or telemedicine provider. | 1,418,146 (12.3) |  |
|  | **MSG10** Watch for COVID-19 symptoms such as fever, cough, or difficulty breathing. If you develop any of these symptoms or if you start feeling worse, call your medical provider, clinician advice line, or telemedicine provider. | 833,536 (7.2) |  |
|  | **MSG17** Watch for COVID-19 symptoms such as fever, cough, or difficulty breathing. If you develop any of these symptoms, call your medical provider, clinician advice line, or telemedicine provider. | 213,495 (1.8) |  |
|  | **MSG15** As soon as possible, tell your occupational health provider (or supervisor) that you may have been in contact with someone with suspected COVID-19. Tell your occupational health provider (or supervisor) that you may have been in contact with someone with suspected COVID-19. You may be asked to wear a mask over your nose and mouth to protect yourself and those around you.   - Check your temperature twice a day for 14 days. - Watch for COVID-19 symptoms. Learn more about COVID-19 and how you can protect yourself and others on the CDC website. | 21,124 (0.2) |  |

^a^ Conversations were classified as “conversation redirected” when the individual: 1) did not provide consent to use the Self-Checker; 2) did not make any selections after starting a conversation with the Self-Checker; or 3) was age-ineligible for using the Self-Checker according to the relevant CDC guidance at the time. Age cutoffs changed during the study period as a result of changes in CDC guidance.

^b^ Conversations were classified as “seek care or contact a healthcare professional immediately” when the individual: 1) indicated having life-threatening symptoms or a possible medical emergency; 2) had a medical condition that put them at risk of becoming more seriously ill; or 3) was in close contact or exposed to someone with COVID-19 in a healthcare or caregiving setting (e.g., care center, nursing home, or homeless shelter).

^c^ Conversations were classified as “take no action, or stay home and self-monitor” when the individual: 1) was not feeling sick and had no symptoms; 2) was feeling sick and experienced symptoms other than primary COVID-19 symptoms; 3) was not feeling sick but may have had exposure or close contact with someone who was diagnosed with COVID-19.

Table A4. Self-Checker Version History – Documented by the Self-Checker Development Team

| **Date-From** | **Date-To** | **Self-Checker Version Released** | **Summary of Changes Made to the Self-Checker** |
| --- | --- | --- | --- |
| 3/16/2020 | 3/23/2020 | 50 (3/19/2020) | - Soft launch of the Self-Checker |
| 3/23/2020 | 3/30/2020 | 51 (3/28/2020) | - Update to underlying health conditions - Updated decision tree logic |
| 3/30/2020 | 4/6/2020 |  | - Introduction of simplified Chinese, Korean, Spanish, and Vietnamese versions of the Self-Checker |
| 4/6/2020 | 4/13/2020 | 53 (4/7/2020) | - Minor language changes |
| 4/27/2020 | 5/4/2020 | 58 (5/1/2020) | - Added asymptomatic flow (care messages, HCW PPE question) - New question on quality of user’s breathing - New COVID-19 testing messages - Added age stratification (≥64 and 65+) on care messages - Consolidated ED/911 care messages - Updated symptom-related questions - Updated care messages - Update to symptoms logic |
| 5/4/2020 | 5/11/2020 |  | - Updated non-English language version of Self-Checker to meet version 58 requirements |
| 5/25/2020 | 6/1/2020 |  | - Updates to non-English language of Self-Checker |
| 6/1/2020 | 6/8/2020 | 60 (6/5/2020) | - Removing pediatric flow (<18 years old) - Updated exposure question to remove "area where COVID is widespread" - Updated symptoms question (no new symptoms, updated verbiage) - Updated testing messages; include eligibility for testing (anyone with COVID symptoms) - Plain language updates - Streamline decision tree to simplify flow - Consolidated two questions about ED/911 symptoms into a single question and moved up in the tree - Updated care messages with hyperlink to HHS website for finding telehealth providers: <https://telehealth.hhs.gov/> |
| 6/8/2020 | 6/15/2020 | 61 (6/8/2020) | - Updated a testing care message to provide hyperlinks to HHS websites for finding a testing location:   <https://www.hhs.gov/coronavirus/community-based-testing-sites/index.htm>  <https://www.hhs.gov/coronavirus/testing/index.html> |
| 6/22/2020 | 6/29/2020 |  | - Updated non-English language version of Self-Checker to meet Version 61 requirements |
| 7/6/2020 | 7/13/2020 | 61.1 (7/8/2020) | - Bug fix to non-English version of version 61 |
| 7/13/2020 | 7/20/2020 | 62 (7/13/2020) | - Update to the list of underlying conditions based on updated CDC recommendations |
| 9/7/2020 | 9/14/2020 | 63 (9/10/2020) | - Added pediatric flow for ages 2 to 17 years   - Includes specific questions for pediatric age groups and settings - Updated COVID-19 testing and care messages and logic - Added a new Care Message |
| 12/7/2020 | 12/14/2020 | 64 (12/10/2020) | - Added new question about testing and results - Added new question about symptoms severity - Added new care message for individuals who have been in close contact with someone with confirmed COVID-19 - Updated 15-minute rule to reference cumulative total time - Revised multiple existing care messages to add additional information - Added new testing messages - Revised testing message to add additional information |
| 12/14/2020 | 12/21/2020 | 65 (12/17/2020) | - Added Headache as a symptom - Added Influenza testing message |
| 12/21/2020 | 12/28/2020 | 65.1 (12/23/2020) | - Removed two questions about symptom severity |
| 2/1/2021 | 2/8/2021 | 66 (2/2/2021) | - Updated testing and care recommendations for pediatric individuals (Age 2 to 17 years) with primary and secondary symptoms of COVID-19 - Added a question on testing for symptomatic pediatrics (Ages 2-17) - Updated demographic questions:  - Added new question on ZIP code  - Added two questions on race and ethnicity  - Revised gender identity question to be   more inclusive   - Down syndrome as a COVID-19 high-risk condition for adults and children - Modified description used for cyanosis in the list of life-threatening conditions |
| 3/1/2021 | 3/8/2021 | 66.2 (3/3/2021) | - Adjusted logic and added messaging around only “Other symptoms” (applies to 2 questions in the adult pathway and 2 questions in the pediatric pathway) - Simplified care and testing messages - Removed 3rd person pronouns - Revised care message to include recommendation to isolate for 10 days until symptoms resolve - Updated introduction messaging - Added endcap message - Moved CDC hyperlinks to end of assessment - Classified rash as “new” in pediatric symptom list |
| 4/19/2021 | 4/26/2021 | 67 (4/19/2021) | - Updated intro and endcap message to include a recommendation to get a COVID-19 vaccine - Introduced question that address the short and long-term effects of COVID-19 - Expanded testing scenarios to account for testing within 10 days to 1 year - Added question to Adult Asymptomatic pathway about testing status in the last 14 days - Revised 3 care messages to account for longer isolation periods for those with a weakened immune system - Revised two testing care messages - Added new line of care and testing messages to be used for long covid pathways - Removed “vaping” from questions - Added note to 1 question about responding to Self-Checker questions for someone else |
